# Young People’s Experiences With an Empowerment-Based Behavior Change Intervention to Prevent Sexual Violence in Nairobi Informal Settlements: A Qualitative Study

**DOI:** 10.9745/GHSP-D-21-00105

**Published:** 2021-09-30

**Authors:** Anna E. Kågesten, Phoene Mesa Oware, Wendy Ntinyari, Nickson Langat, Benjamin Mboya, Anna Mia Ekström

**Affiliations:** a Department of Global Public Health, Karolinska Institutet, Stockholm, Sweden.; b Institute for Social Development, University of the Western Cape, Cape Town, South Africa.; c Ujamaa-Africa, Nairobi, Kenya.

## Abstract

**Purpose:**

Young people in sub-Saharan Africa face one of the world’s highest burdens of sexual violence. Previous impact evaluations indicated that a 6-week empowerment-based behavioral intervention in Nairobi informal (slum) settlements can reduce sexual assault. This qualitative study investigated girls’ and boys’ experiences of the intervention to identify potential mechanisms of change.

**Methods:**

We conducted a qualitative study in Nairobi slums with students (aged 15–21 years) who had participated in 2 parallel school-based curriculums called IMPower (girls) and Your Moment of Truth (boys) at least 1 year ago. Data were collected via 10 focus group discussions (5 for boys, 5 for girls) with 6–11 participants in each and 21 individual in-depth interviews (11 boys, 10 girls) that explored participants’ experiences of the intervention and their suggestions for improvement. Findings were analyzed using thematic network analysis guided by empowerment theory.

**Results:**

Girls described how the intervention enabled them to recognize and resist sexual assault via verbal and physical strategies for self-protection, negotiate sexual consent, and exercise agency. Boys described increased ability to avoid risky behaviors and “bad” peer groups and to understand and respect consent. Girls also described how the intervention strengthened their self-confidence, and boys said that it boosted positive life values and gender-equal attitudes. Skilled facilitators and interactive and relevant content were highlighted as key to intervention success. Areas of improvement included expanding the curriculum to contain more content on sexual and reproductive health and rights and involving out-of-school youth, parents, teachers, and communities.

**Conclusion:**

Findings indicate that a relatively short, behavioral school-based intervention can empower both girls and boys to prevent various forms of sexual violence in a low-income setting where it is endemic. Incorporating multilevel support structures, such as involving communities and families, could further enhance young people’s long-term safety, health, and well-being.

## Abstract (precis)

This study indicates that an empowerment-based, behavioral intervention can contribute to equipping both adolescent girls and boys with concrete skills to recognize and resist sexual violence and can promote positive, nonviolent masculinities among adolescent boys.

## Teaser

**Key Findings:**

- A 6-week behavioral, school-based intervention can contribute to empowering adolescent girls to recognize and resist sexual violence and to exercise agency.
- The intervention can also promote positive, nonviolent masculinities among adolescent boys and encourage rejection of harmful stereotypes.
- Skilled, thoroughly trained local facilitators and interactive, adolescent-friendly relevant content were highlighted by the adolescent participants as key to intervention success.

**Key Implications:**

- Policy makers should consider integrating empowerment-based, behavioral interventions in standard school curriculums to prevent sexual violence and harmful gender norms.
- Policy makers should adopt legislation that will facilitate the implementation of comprehensive sexuality education that addresses topics such as sexual consent communication and guarantees access to adolescent-friendly sexual and reproductive health services.
- Public health practitioners and implementers should consider expanding similar interventions to include comprehensive sexuality education as well as directly targeting the broader social environments in which adolescents are socialized.
- More research is needed to better understand the longitudinal effects of the intervention and the strategies for sustainable implementation in different contexts.

## INTRODUCTION

Gender-based violence (GBV) remains a major global public health problem and human rights violation.^1^ Globally, 1 in 3 women reports experiencing physical and/or sexual violence in her lifetime.^2^ Multiple, well-established consequences for this violence exist in regards to sexual and reproductive health,^3^ physical and mental health,^4^ and social and economic development.^5^ GBV, including sexual violence, begins early in life,^6^ with an estimated 1 in 10 girls (120 million) having been forced to perform sexual intercourse or other forms of sexual acts before her 20th birthday.^7^ Likewise, according to a 4-country study in sub-Saharan Africa, between 8% and 27% of boys and young men aged 13–24 report ever perpetrating sexual violence,^8^ and a global study in poor urban settings found that up to 1 in 10 boys aged 15–19 years reported perpetration of sexual violence against an intimate partner in the past year.^9^ Thus, focusing on adolescence as a formative period for shaping attitudes and behaviors linked with both violence exposure and perpetration is both a research priority^10^ and central to achieving gender equality in line with Agenda 2030.^11^

Young people in Kenya face one of the world’s highest rates of sexual violence and other forms of GBV. National data indicate that 35% and 47% of young women (aged 15–19 and 20–24 years, respectively) have experienced physical and/or sexual violence in their lifetime. Husbands are the most common perpetrators of sexual violence for married women, whereas, for never-married women, the perpetrators are commonly a stranger, friend, or acquaintance.^12^ The risk of GBV is especially high in urban informal settlements (slums), which are characterized by high levels of poverty, unemployment, violence, crime, and a lack of health and educational services. According to a recent survey in 2 Nairobi informal settlements, 33%, 23%, and 16% of young women report experiencing psychological, physical, and sexual violence, respectively, in the past year.^13^

In Kenya, sexual violence and other forms of GBV are driven by multiple social-ecological factors,^14^^,^^15^ including gender norms that undermine the power of girls and women and expect boys and men to adhere to stereotypical masculinity ideals of violence and risk.^15^ Empowerment self-defense (ESD) is an interactive feminist training to prevent sexual violence by building verbal, emotional, and physical skills to recognize and resist different forms of GBV through heightened self-esteem and confidence.^16^^–^^18^ Previous studies in high-income settings indicate that ESD is a promising strategy for reducing sexual assault and coercion against female students on college and university campuses.^17^^,^^19^^,^^20^ In low- and middle-income countries, ESD has primarily been used in primary and secondary schools via an adapted curriculum called IMPower, developed by the U.S.-based nongovernmental organization No Means No Worldwide.^16^ In Kenya and Malawi, IMPower is implemented in Nairobi slum schools by the local nongovernmental organization Ujamaa-Africa, with cluster-randomized evaluations indicating reduced incidence of past-year sexual assault among girls aged 10–19 years.^16^^,^^21^^,^^22^

As global scholars have noted, the responsibility for GBV ultimately rests with the perpetrator.^23^^,^^24^ Thus, a comprehensive approach to sexual violence prevention must address underlying gender norms and support the development of nonviolent behaviors among young men as agents of change in bolstering gender equality. In Kenya, IMPower has therefore been combined with a parallel and complementary curriculum called Your Moment Of Truth (YMOT) that works with boys and young men to build positive masculinities and skills for verbal bystander intervention.^25^^,^^26^ Even though YMOT’s effect on sexual violence perpetration has not been evaluated as part of peer-reviewed research, a 2015 evaluation in Nairobi slums found that boys who received the intervention were more likely than the control group to hold gender-equal attitudes and to intervene successfully when witnessing GBV.^26^

To date, no qualitative research has explored if, how, and under which circumstances girls and boys can draw on skills taught by these curriculums to prevent sexual violence. Such research could help provide important insights into the potential mechanisms of change that underlie the intervention in a way that is not elucidated in survey research. In light of findings from a 2020 review conducted by the What Works to Prevent Violence Against Women and Girls Global Program, which indicated that more evidence is needed on the intervention given limitations in previous study designs,^27^ qualitative research can help to better understand participants’ experiences.

In response to this gap, the current study aims to understand girls’ and boys’ experiences of this empowerment-based behavioral intervention in schools to prevent sexual violence in Nairobi slums. Focusing on changes in perceived agency and empowerment, we seek to elucidate both positive aspects of the intervention as well as areas for improvement.

The current study aims to understand girls’ and boys’ experiences of an empowerment-based behavioral intervention to prevent sexual violence in Nairobi slums.

## PROGRAM DESCRIPTION

Both the intervention for girls (IMPower) and boys (YMOT) were developed by No Means No and Ujamaa-Africa for the East African context via extensive formative work, including focus groups and piloting.^22^^,^^26^ Both curriculums are implemented simultaneously over a 6-week period and comprise 5 2-hour sessions that are taught in separate complementary classes, followed by a sixth joint session. The girls’ curriculum (IMPower) uses ESD techniques to strengthen girls’ critical reflection and problem-solving skills and to boost their self-esteem and confidence. The curriculum also offers hands-on risk-reduction techniques for recognizing and resisting different forms of sexual harassment and violence, such as boundary setting, diffusion tactics, verbal assertiveness, and negotiation (e.g., name potentially threatening behaviors from abusers), and if needed, different forms of physical self-defense (e.g., bodily weapons) as the last resort. Throughout the intervention, girls disclosing violence or abuse are connected with the Sexual Assault Survivors Anonymous program at each site. The boys’ curriculum (YMOT) is designed to promote positive, nonviolent masculinities and to help boys identify emotions and build skills for nonviolence, seeking consent, and strategies for safe bystander intervention (i.e., interrupting potential violence and harassment).

**Figure:**
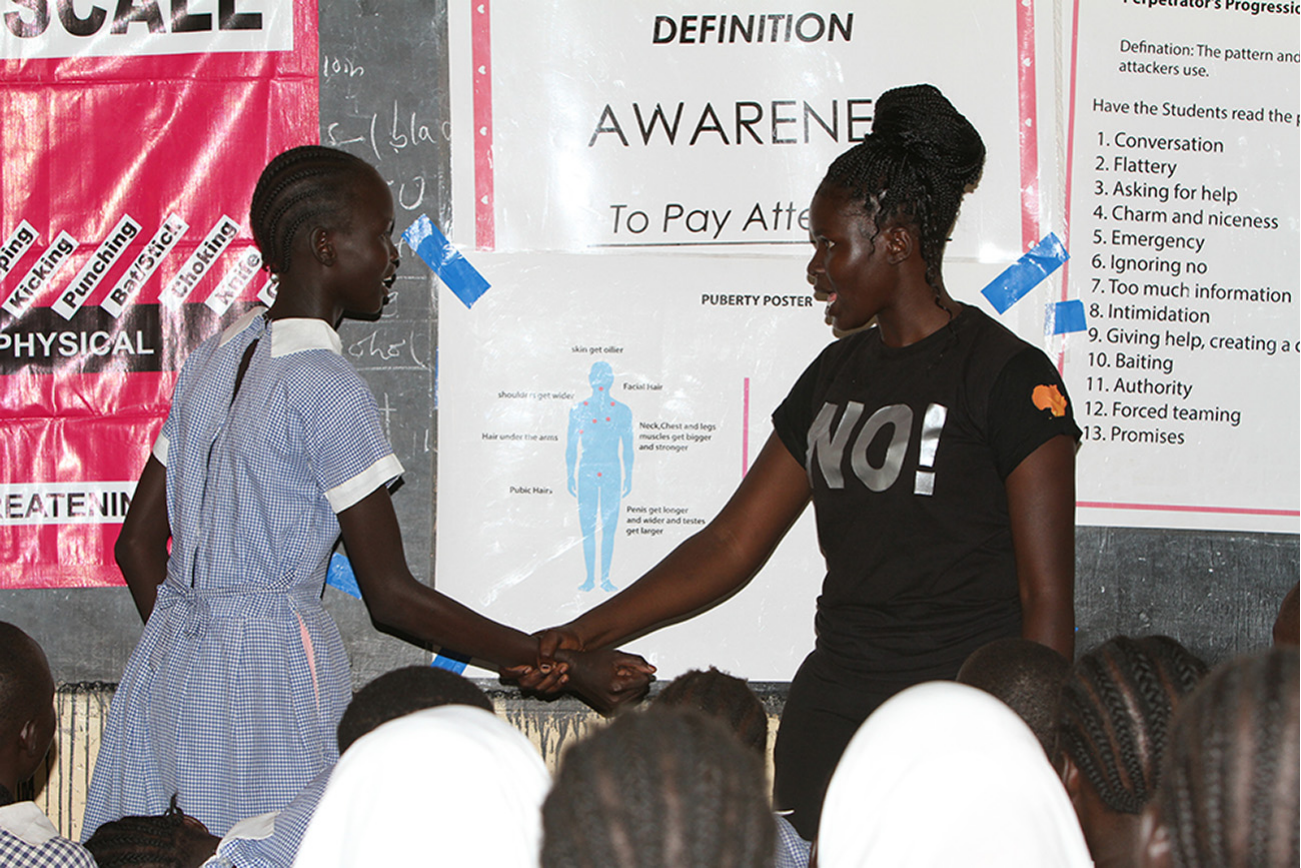
A female student practicing the wrist grab release in front of her class. © 2018 Peter Omondi/Ujamaa Africa

The sessions are designed for ages 10–19 years, but the specific content is age-adapted. A 2-hour refresher session, which is typically attended by at least 80% of program beneficiaries, is given at 6 months and at 1 year after training. Both curriculums are highly participatory and include facilitated discussions, critical reflection, role-play, and drama. Table 1 provides a detailed description of each session.

**TABLE 1.** Intervention Curriculum Themes, With Age-Adapted Content

**Girls: Empowerment Self-Defense (IMPower)**	**Boys: Your Moment Of Truth (YMOT)**
**Session I: Introduction** Introduction to the program and girl’s empowerment self-defense, covering a broad range of safety options and goals, including the Assault Continuum, a tool used throughout the intervention to demonstrate different forms of violence ranging from verbal harassment to rape.	**Session I: Introduction** Introduction to the program with facilitators sharing personal stories about important “Moments of Truth” in their lives. The goal is for boys to develop awareness about their own important moments of truth that they might face.
**Session II: Verbal empowerment skills** Verbal skills to prevent violence, including saying “no” in a way that is effective and believable, showing assertiveness, setting boundaries, lying to or tricking potential assaulters, and making a scene to alert others to aggressive acts and behaviors. Understanding how awareness and paying attention can be a powerful line of defense.	**Session II: Skills to prepare for YMOT** Highlighting skills for awareness, how to identify red flags in potential assault situations, and how to use assertive body language and verbal response. Discussion around courage based on personal stories from facilitators and use of role-play to deter physical violence.
**Session III: Physical empowerment skills (I)** Girls are introduced to thinking about their bodies as powerful and equipped with potential tools or weapons (hands, feet, other body parts) that can be used for self-defense against an attacker. Facilitators use role-play to demonstrate scenarios, using concepts such as “What’s Free, What’s Open” where girls learn and practice ways to escape and holds and grabs that minimize power differentials.	**Session III: Bystander intervention** Definition of bystander intervention to interrupt (potential) violent situations in a safe way. Facilitators demonstrate skills using role-play, followed by boys practicing stepping up and trying to recognize or resolve potential conflict.
**Session IV: Physical empowerment skills (II) and disclosure** Focus on self-defense in more extreme situations, with facilitators using mats and strike bags to simulate attack situations and defense. Introduction to disclosure and the importance of telling someone about gender-based violence experiences, and to counter victim blaming. Girls who disclose assault to facilitators are directly linked with the Sexual Assault Survivor Anonymous (SASA) support service.	**Session IV: Sexual consent** Definition and understanding of sexual consent, with facilitators and boys discussing what “counts” as consent, exploring the causes of and myths about rape, and covering basic de-escalation and negotiation techniques.
**Session V: Advanced empowerment self-defense** Skills to de-escalate violence by using language that can help when someone known or a stranger is upset or becoming upset. Girls practice stalling an attacker by using a one-liner that keeps the assailant calm while they plan their next move.	**Session V: Responsibility for one’s self** Boys discuss what it means to be responsible for one’s actions and behaviors and continue practicing utilizing bystander intervention skills.
**Session VI: Putting it all together** Review of key concepts, with girls continuing to practice verbal and physical skills to identify and escape violent/threatening situations and discuss when and how to disclose violence.	**Session VI: Review of skills and content** Review of key concepts, practicing skills via role-play. Boys make public commitments to utilize their new skills and to face their moments of truth.
**Integrated class for both boys and girls** Creates a platform to discuss cross-cutting issues including understanding harmful gender stereotypes, basic instances that require consent, and gender roles and the impact they have on both males and females.	

The facilitators are carefully selected from the local communities, must be aged 20–30 years, and have at least 2 years’ experience in GBV prevention among young people. Following interviews, selected facilitators undergo a 3-month, 250-hour training by expert instructors, which ends with a rigorous examination, as well as practical demonstrations in a class with adolescents. New facilitators teach alongside an experienced instructor for the first year before facilitating groups independently.

### Theory of Change

The underlying theory of change behind the combined curriculums is that girls who have a sense of control over their bodies and a belief in their rights are more likely to resist sexual violence and to negotiate sex in (wanted) intimate relationships. Furthermore, working with young men to critically reflect on gender norms, consent, and attitudes towards and involvement in violent behaviors is central to reduce perpetration. For the current study, we use VeneKlasen and Miller’s^28^ empowerment framework to understand and organize participants’ experiences in light of the theory of change. The framework explains how 4 different manifestations of power affect GBV, with *power over* (an individual/group) referring to the ability of someone (e.g., perpetrators) to dominate others. We hypothesize that the intervention helps to reduce power over by strengthening girls’ and boys’ *power to* protect themselves and others; their *power within* (e.g., self-confidence, self-efficacy, sense of self-worth); and their *power with*, a collaborative form of power that is achieved through building bridges between individuals to achieve a common goal (e.g., to end GBV in their communities).

We hypothesize that the intervention helps to reduce *power over* by strengthening girls’ and boys’ *power to* protect themselves and others; their *power within*; and their *power with*.

## METHODS

### Study Design and Participants

We conducted a qualitative study with former intervention participants at 5 schools in 4 Nairobi slums: Kibera, Dandora, Mkuru-Viwandani, and Huruma. Purposive sampling was used to invite participants who were at least 15 years old and who participated in a minimum of 4 curriculum sessions at one of the selected schools, at least 1 year before data collection. Recruitment was performed by Ujamaa-Africa’s field staff, aided by the research team. Participants were first screened for eligibility by filling out forms in which they indicated interest and availability to participate in either focus group discussions (FGDs) or in-depth interviews (IDIs). When more students than needed were eligible and interested at each school, all papers were put into a closed box from which the study staff made a random selection.

### Data Collection

Data were collected via a series of FGDs and IDIs in English, Kiswahili, or Sheng (local slang) between January and June 2019. We conducted a total of 10 FGDs (5 with boys, 5 with girls) with 6–11 participants in each group and 21 IDIs (10 girls, 11 boys) (Table 2). Most participants came from schools in Dandora and Mukuru-viwanndani due to the ease of accessibility of eligible participants from these sites in school settings. Because adolescents living in Nairobi’s informal settlements face similar sexual violence risk factors^29^ and because an examination of any potential variation or similarities in participants’ experiences of the intervention by location within Nairobi is beyond the scope of the current study, the uneven representation of study participants by location was not viewed as compromising the findings. Interviews were conducted in person by trained data collectors employed by Ujamaa-Africa who had not been part of delivering the intervention but were local to the study area and spoke the language(s). All data collectors underwent a 1-week training in qualitative methods led by experienced researchers at Karolinska Institutet during October 2018, followed by a 4-day refresher training in January 2019. The training covered qualitative methodology, ethics, recruitment, interviewing skills, reflexivity, fieldwork logistics, and transcription.

**TABLE 2.** Number of Interviews, by Location, Method, and Gender

**Location**	**Gender**	**Focus Group Discussions**	**In-Depth Interviews **
Kibera	Boys	1 (8 participants)	2
	Girls	1 (8 participants)	1
Dandora	Boys	1 (11 participants)	4
	Girls	1 (10 participants)	5
Mukuru-viwanndani	Boys	2 (8 participants)	4
	Girls	2 (8 participants)	4
Huruma	Boys	1 (11 participants)	1
	Girls	1 (9 participants)	0
Total	Boys	5 (46 participants)	11
	Girls	5 (43 participants)	10

A set of semi-structured interview guides (piloted in 2 FGDs and 2 IDIs before implementation) were tailored to the type of interview (FDG vs. IDI) and participants’ sex, and covered research questions related to participants’ overall experiences of the program, impact on sexual violence, agency, gender norms, sexual relationships, and suggestions for improvement. FGD guides were designed to elicit responses around commonly held views on subjects such as gender norms and participants’ shared perceptions and experiences of the intervention, while IDIs provided a more private environment for participants to open up about personal experiences including violence. Participants’ memorable events were used to probe for more details on if, and how, they had applied the skills in real life. Participatory methods were used to engage participants, drawing on techniques such as a word-cloud activity (asking participants to describe themselves before and after the intervention) and photo-elicitation (showing pictures of pregnant girls at school with questions around potential causes, consequences, and prevention).

Participants related their overall experiences with the program and its impact on their experiences of sexual violence, agency, gender norms, and sexual relationships.

Data collection began with FGDs to update and refine the interview guides and identify themes for further exploration in IDIs. All interviews were matched by gender, with female data collectors interviewing girls and young women and male data collectors interviewing boys and young men, and took place in a private space to ensure participants’ confidentiality and privacy. Note-takers took extensive field notes during FDGs, and all data collectors wrote reflective notes after each interview to summarize key themes and context details. Interviews were audio-recorded, transcribed verbatim in Kiswahili/Sheng, and translated into English.

### Data Analysis

Data were analyzed using thematic network analysis,^30^ which includes assigning labels (codes) to text segments in the transcripts, followed by the identification of code and categorization into *basic themes*. These were further clustered into categories called *organizing themes* and broader *global themes* that best conveyed the meaning of the data concerning the research questions explored. Transcripts were uploaded into Atlas.ti (Scientific Software, version 8), followed by deductive as well as inductive coding by 2 researchers (PMO, AK) to create an initial codebook applied to the remaining transcripts, adding new codes as needed. Themes and subthemes concerning the study aims were organized according to the VeneKlasen and Miller empowerment framework.^28^ We used several strategies to enhance the credibility of our findings, including double-coding and recoding of transcripts to ensure consistency as well as member checking to confirm emerging themes with interviewing staff.

### Ethical Considerations

Ethical approval was obtained from the National Commission for Science, Technology, and Innovation (NACOSTI) in Kenya. Written informed consent (assent for minors) was sought from all participants. Parental or guardian consent for minors was waived by the ethics board due to the sensitive topics explored, as children might be uncomfortable asking their parents’ permission if it may increase their risk of violence and/or affect their ability to seek services.^31^ Other protection mechanisms were used to ensure the safety, confidentiality, and privacy of minors, including parental opt-out forms (allowing parents/guardians to object to the participation of their child in the study) and school headteachers witnessing the assent process. In case of violence disclosure, participants were referred to local services for sexual assault survivors. All participants were also provided with written contact information to local child protection and support services, irrespective of violence disclosure or distress.

## RESULTS

The Figure presents a framework of how the intervention works with girls and boys to prevent different forms of sexual violence based on their experiences and according to the themes identified. Overall, our findings indicate several potential mechanisms of change, including that the intervention helped to strengthen the power to recognize and resist unwanted sexual experiences, communicate sexual consent, and exercise agency among girls and to reduce risky behaviors, avoid “bad” peer circles, and understand and respect sexual consent among boys. Participants’ stories also highlighted how the intervention helped them to strengthen their power within—in the form of self-confidence and self-awareness for girls and positive life values and rejection of harmful masculinity norms for boys—and boosted power with their communities (parents, friends, and teachers). Below, we describe girls’ and boys’ experiences (using pseudonyms) concerning the different dimensions of power. By including both positive and negative or challenging aspects of the intervention, we reflect on essential implementation elements and end with a summary of participants’ suggestions for improvement.

**FIGURE:**
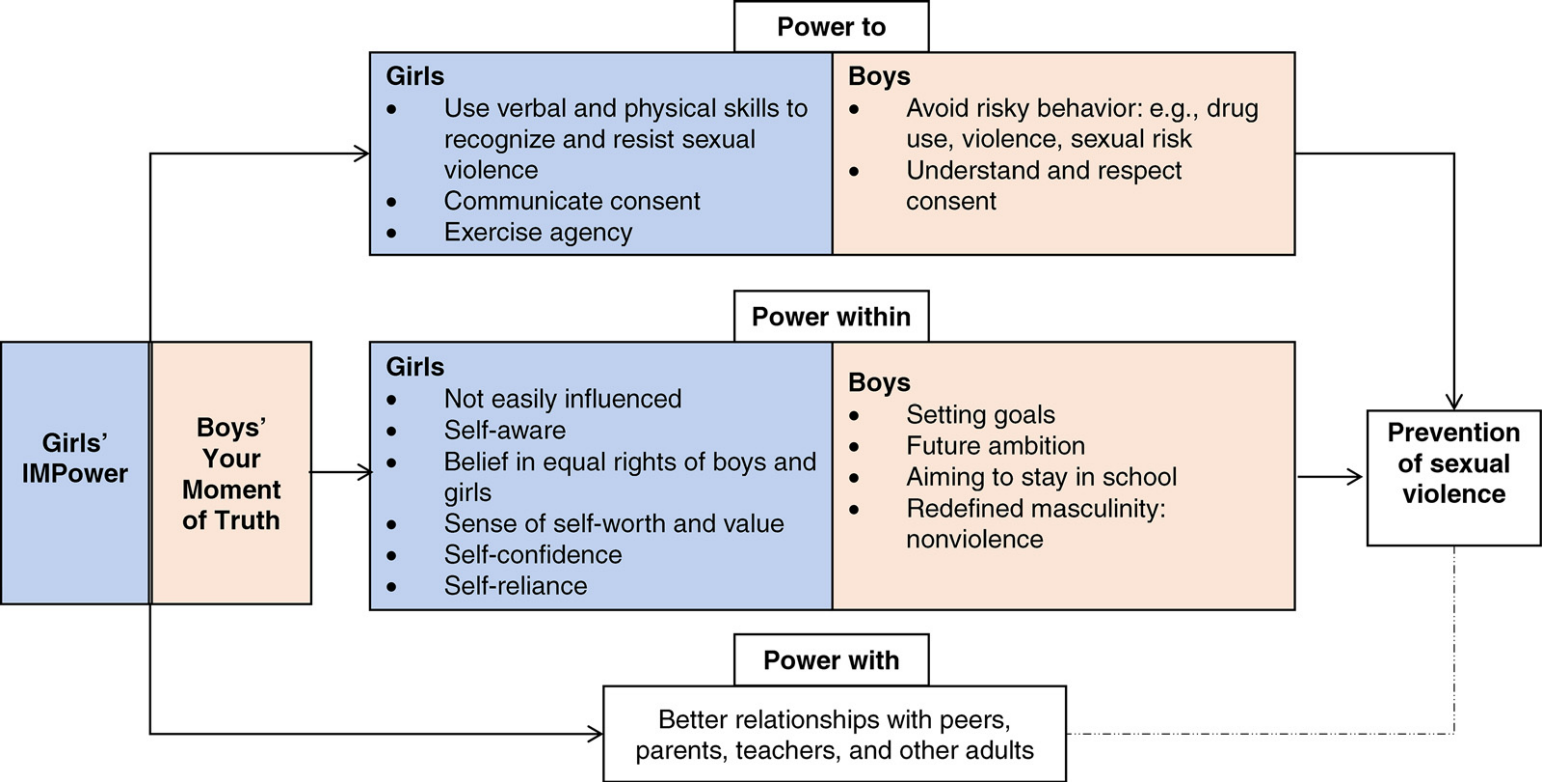
Themes Identified Related to Potential Mechanisms of Change Through Which the Combined Intervention Curriculums May Empower Girls and Boys to Prevent Sexual Violence

Several potential mechanisms of change were observed, and participants’ stories highlighted how the intervention helped them to strengthen their power within.

### Power To

Girls described and demonstrated a range of physical skills that they remembered from the curriculum training, such as bodily target areas and using body parts as weapons. They also described psychological skills such as tricks, negotiation, causing a scene to draw attention, de-escalation of potentially violent situations, and consent communication. Recalling situations in which they used curriculum lessons, girls’ narratives showed that, by using these skills, they possessed the power to stop sexual assault. Girls described walking in slum alleys to be a particularly vulnerable experience, noting how, for example,

*In the hood now it’s even worse, there like you are just passing, sometimes you find the boys seated there, they block your path yes, then they take turns pulling you and you are left hopeless.* —Nuru, female IDI participant

Experiences such as those in the following description aptly demonstrate the use of skills from the curriculum to both recognize and de-escalate such potentially threatening situations.

*You know in the slum, everywhere, there are people sitting everywhere. (…). Now the people who sit there, usually smoke “bangi” [marijuana]. When you walk past, you hear them say “niaje” [informal greeting, commonly used by youth] (…) if you fail to respond, they all come and stand in front of you. Now, if you act like you want to run away, they will tell you, “We too can run.” So you have to stop. So, for me … it has happened twice. It was two alleys that I used and experienced something of the sort. I stopped. They said, “Habari”—“mambo” [what’s up]. I replied “poa” [cool]. Now, when I stopped, they were startled, they have never seen anyone stop. Most people usually run. I stood. Because there is nowhere you can run to. And I had been told in the [IMPower] training if you notice that someone is big, bigger than you, or if there are many people [potential attackers], you need to first calm down, concentrate, then find out what they want and where they are going (…) So I stopped. (…) after I responded to all of their questions, they let me be.—*Rhoda, female IDI participant

Girls also told stories of speaking up against unwanted attention and touching, saying that the intervention enhanced their capacity to exercise agency to protect and advance their interests in life. They described being firm and forceful and maintaining eye contact whenever they said “no” to someone whose attention was unwelcome.

*I have an uncle who used to come to our place (…) he used to pretend it’s playing games with us but instead he is touching one’s breast. So he used to touch me and I became annoyed but there was nothing I could do because he was huge. Now I—this day that we were taught those things by [IMPower], we were taught how you can use your voice and nowadays when he tries to touch me I become harsh, and tell him no with a loud voice, (…) with confidence I tell him “Leave me!” and “Do not try to touch me.”* —Emily, female FGD participant

*Before we were taught [IMPower] my “no” was not as firm, it was a weak “no.” So now after learning this we learned how special our bodies are and nobody should mess with them, so now when you say “no” it means exactly that.* —Nuru, female IDI participant

Examples of girls forcefully saying “no,” like the ones illustrated above, were often limited to encounters outside the context of steady relationships. With intimate partners, communicating and negotiating consent was sometimes more vague and complicated, with both boys and girls differentiating between a “hard” and “soft” no.

*Because there is a “no” that shows that one is serious, yes, “NO!” and there are those that say “no” as they draw on the ground, they say “no” [lowers tone]. So, the way I see it, there are two “no’s.” The “no” that shows that you don’t want, serious. You put on a stone face, meaning you don’t want that thing. —*Zawadi, IDI female participant

*I have never been told “no,” but it depends on how that girl says it. When she says it in a low voice, you should know that that “no” means “yes.” But when she stands up and says “No, I don’t want!” in a loud voice, it means that she is serious about rejecting that thing.* —Luke, male FGD participant

Narratives from both male and female participants illustrated a double standard of consent communication in intimate relations, with “no” needing to be qualified, for example, by nonverbal cues versus in unwanted or platonic relations, in which “no” *really* means “no.” While boys explained that the training had helped them seek and understand sexual consent, they did not automatically interpret a “no” within intimate relationships as simply meaning “no.” For example, boys narrated how it was difficult to believe that a girl was serious about their “no” the first time, but that the training had nonetheless taught them to seek consent and respect (persistent) refusal:

Narratives from both male and female participants illustrated a double standard of consent communication in intimate relations, with “no” needing to be qualified.

*I wouldn’t believe her at first. If she told me “I don’t want,” I would tell her, “You are just kidding me.” You will eventually give in. But if she refuses, I will respect that, I will tell her it is okay. Because we were taught by Ujamaa that if a girl says she’s not interested, tell her it’s fine. Leave her alone.* —Faraji, male IDI participant

Stories from male participants further highlighted how the boys’ curriculum works to increase their power to stay away from or quit risky behaviors, such as violence and drugs, that act as risk factors for violence perpetration. After undergoing the training, boys described not only disapproving of violence but also attempting to refrain from it in their daily lives. They further explained how the training had influenced their capacity to make better friendship choices, to leave “bad company” and/or resist peer pressure, and abstain from using drugs altogether. In one IDI, the interviewer asked a participant whether he could talk about a time when he applied what he was taught. The participant responded,

*Bad company. Because I stopped associating myself with, friends who, those friends who, who only think of evil things. And then also… using drugs. I mean, I have never indulged in drugs but [the training] has helped me know the dangers, I mean. . . . It has helped me learn more about drugs. —*Francis, male IDI participant

**Figure:**
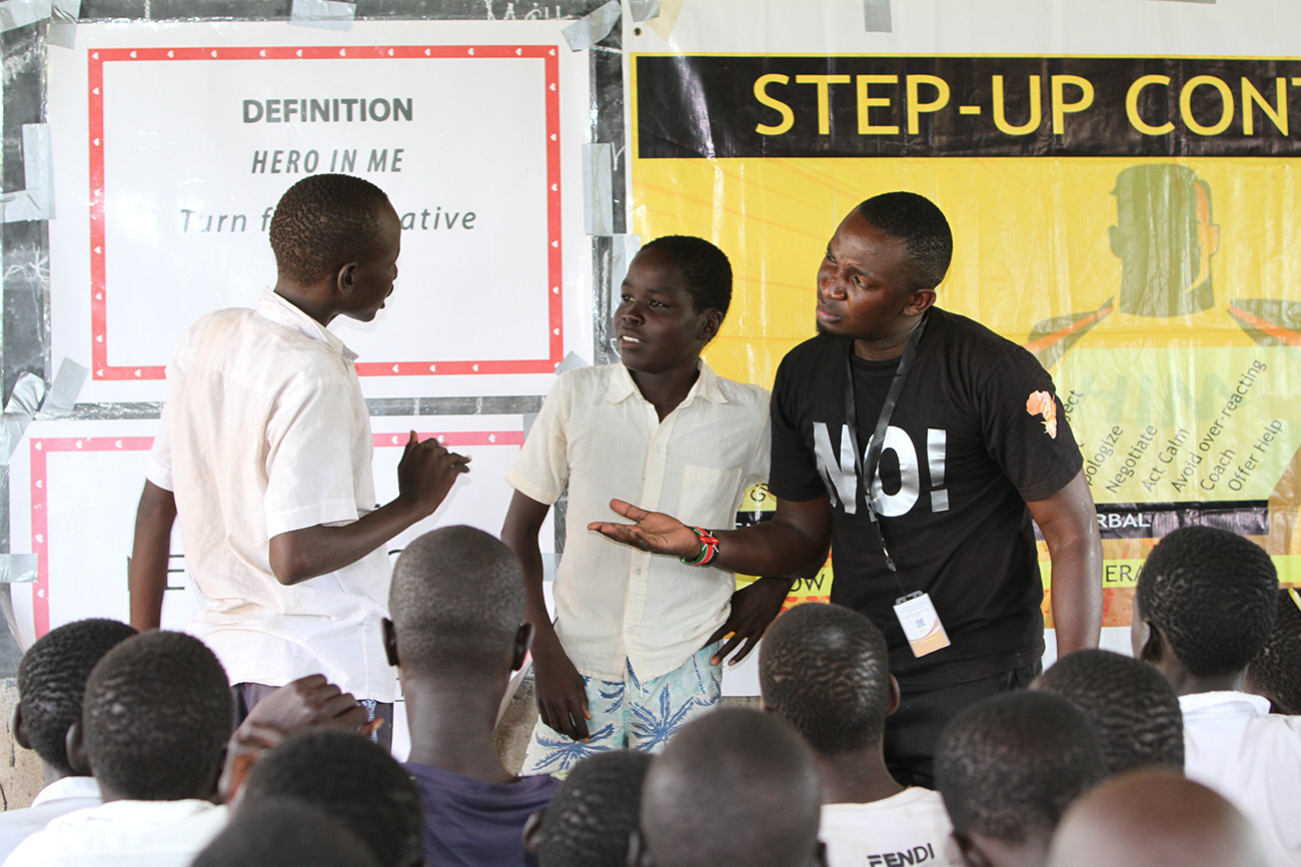
An Ujamaa male instructor leading primary school male beneficiaries in a de-escalation skit. © 2018 Rose Alice/Ujamaa Africa

The training thus not only encouraged young boys to abstain from vices such as drug use and bad company, but it also appeared to affirm the positive choices that some male participants had already made.

For boys living in slums, resisting peer pressure and choosing to walk away from bad company could be challenging, however, and several male participants described how alternative choices could result in isolation, stigma, and sometimes threats.

For boys, resisting peer pressure and choosing to walk away from bad company could be challenging, while girls experienced negative responses when standing up for themselves.

*I have lost my friends in school because that day when we were going home, they asked me to join them to do drugs and I refused. They began calling me a snitch because I joined [Ujamaa], but now I am proud (…) So even at home, many were angry with me because I refused to do the things that they are doing.* —Nathan, male FDG participant

Despite experiencing some challenges, boys were nonetheless proud of the choices and changes they had made, stating:

*Not following people. I am not their shadow. When they do something, I do it too. So I do my own things, and life is good*. —Ali, male FGD participant

or acknowledging potential pitfalls which they had avoided,

*I am very happy because if I would not avoid, I would be in a bad place*. —Jesse, male FGD participant

Similarly, applying the learned skills did not come without challenges for girls, as illustrated by one female participant who spoke up against a boy harassing her in class:

*There are challenges and also good things [with what we learned] because there is a boy in our class, he likes (…) disturbing me, so one day he wanted to touch my breast and I told him, I shouted at him and told him to leave me alone, I shouted at him in front of the class, he felt ashamed in front of them, [and] he began to abuse me in front of them (…) he insulted me with all names (…) I felt bad but [it] also helped me because even those other boys saw I was not that “less” [meaning, not too soft to be intimidated] (…) if they came to me they know I am “fire.” And they can’t joke with me. —*Emily, female FGD participant

While Emily was verbally harassed for speaking up against the violence that was directed at her, she also described feeling empowered; her act of speaking up deterred future assaults from her classmates. Another female participant, Rhoda, described feeling guilty about drawing public attention to her assailant, which resulted in him being beaten—yet, she acknowledged that the beating was not her fault:

*I was at the bridge, a man walked past me and he touched me—he touched my breasts. So you know, I was there, I picked up a stone, and threw it at him. He was caught by some people who beat him up. But it was not—I mean—as in—I was not happy as he was being beaten up, but I just let it be because it was his mistake.* —Rhoda, female IDI participant

As exemplified in these quotes, girls who described facing repercussions when using self-protection skills from the curriculum did not appear to blame themselves but perceived these skills as useful and empowering despite not always working as intended.

### Power Within

In terms of power within, girls recounted how the curriculum boosted their ambitions and confidence in their human rights and sense of self-worth. They also described having the agency to act and actualize choices and aspirations that they valued, be more self-reliant and assertive, and exercise agency in relation to others.

*You know how boys will—they will—as you walk to the back of the class, they will say things like—“ah wewe! wewe!” [“Hey you! Hey you!” while checking a girl out] (…) I recalled what I was told, that we need to defend at least a fellow girl or help someone when they are in need (…) I stood up and told them that what they were doing was not good, we need to live well like sisters and brothers, and we need to help each other. Then they let her be.* —Rhoda, female IDI participant

Boys described how the curriculum helped to instill positive life values, such as self-discipline, confidence, and having life goals. Boys’ narratives further indicated that they disagreed with harmful notions of masculinity, such as sexual aggression, violence, and drug use—emphasizing that a real man should have “self-control,” be “responsible,” “resist peer pressure”, “focus on their education,” and “respect” the rights of women and girls.

Boys described how the curriculum helped to instill positive life values, such as self-discipline, confidence, and having life goals.

*You will find that another boy would hit a girl if she did wrong. At times the boys tell me that I am being too soft with the girls and that because I don’t hit them when they wrong me, I act like one of them [girls]. This forces me to talk to them and explain that it is not necessary to [use] violence when a girl does something wrong to them because beating up the girl does not resolve anything—it only changes their perception towards you. —*Martin, male IDI participant

However, perceptions on femininity remained largely stereotypical, with boys noting that “good girls” should be “shy, polite, kind, loyal,” “clean, humble, and respectful,” and “remain [a] virgin,” as is evident in the views below by 2 male participants in an FGD.

*According to me, first, a good girl is always shy, polite, kind, loyal. . . .she is supposed to be a virgin because she is a girl not a lady/madam … she is supposed to be a virgin, be polite and clean and also be humble and be respectful.* —Shem, male FGD participant

*I’d say, according to me, good girl, as in should behave … like … like you said “be a good girl” should like. . . . should not be doing some things … like … bad things…things like . . . let’s say being a chura [whore] … as in “kupenda hio kitu sana” [love sex too much]. . . . as in such things. —*Charles, male FGD participant

These findings reveal that while the boys’ curriculum may have positively shaped boys’ views of nonviolence and positive masculinities, attitudes about gender norms and the roles of women in relationships appear to be complex and deeply ingrained.

### Power With

Both boys and girls told stories of how the intervention helped them build better relationships with adults such as parents, teachers, and guardians, thereby increasing their “power with.”

Both boys and girls related how the intervention helped them build better relationships with adults, thereby increasing their “power with.”

*I mean from that day [when the training began] I stopped being rude. I started respecting people. I mean—let’s say when a teacher talks to me, I could respond to her/him, unlike how I was before. —*Francis, male IDI participant

Boys further described themselves as positively influencing their peers, demonstrating a ripple effect of the training that potentially extends beyond the individual. In so doing, participants inadvertently created environments that could sustain their positive changes.

*My life changed. You find even how I relate to people—You find that I now have friends. I started changing in that I built my relationship with my teachers. Even at home, when my brother is around, we could relate well, we could have a conversation. If there was something we were supposed to do, we would do it together like people who are familiar with each other*. —Juma, male IDI participant

While there were similar stories among girls, we did not identify any concrete examples highlighting how the curriculum led to increased “power with” for girls that, in turn, enabled them to recognize and resist different forms of sexual violence.

### Essential Implementation Elements: Positive Highlights and Potential Improvements

#### Skilled Facilitators and Relevant, Interactive Content

Both boys and girls emphasized the importance of skilled facilitators, interactive teaching methods, and relevant content. Facilitators were described as being relatable, fun, realistic, and “free,” allowing for self-expression. Girls also highlighted the facilitators’ openness and confidentiality as especially positive, and boys underscored the use of demonstrations with real-life examples in local slang as instrumental.

*It is usually very hard for a boy to express himself when asked a question by the teacher, it is hard to answer the question, it is a must you be in a group of the same gender like now males, so that you can be able to express your points. So when we were there [YMOT training], we were very free. —*Mike, male FDG participant

Participants further described the training as “honest,” “open,” “unique,” and relevant to their daily challenges. Similar to how a male FGD participant noted that “the facilitators were facilitating (…) the reality … the real thing, something that happens … around us,” a female participant highlighted how the teaching methods and content of the program felt especially relevant given the prevailing risk of sexual violence in Nairobi slums.

*For me it was enjoyable because the time that we were being taught those teachings there were so many cases of rape, we were hearing that a girl has been raped in school so it was motivating me to come and hear those ways that I can use to defend myself if it was me … —*Daisy, female FGD participant

#### Areas of Improvement

Participants also discussed several ways to strengthen the intervention curriculums. First, girls and boys alike suggested including additional topics on how to initiate and maintain healthy intimate relationships with partners. For example, Emily, a female FGD participant, stated that the training should focus on “how to manage it (a relationship),” while Francis, a male FGD participant, also explained that:

Participants noted ways to strengthen the intervention curriculums, such as including information on healthy intimate relationships and sexual and reproductive health services.

*… they [the facilitators] should not stop us, they should not tell us that it is wrong to have girlfriends, but they should encourage us on how we can live with girls (…) in a healthy relationship, that you don’t involve yourselves in other things like sex, yes. —*Frank, male FDG participant

Girls pointed out that the subject of contraceptives was missing from the curriculum, and both boys and girls described contraceptives as risky and taboo outside of marriage, highlighting the need to address misperceptions and myths.

*You even find a form 2 girl [approximately 16 years] using family planning and you wonder what for, even the gospel says family planning is for the married, it’s not meant for school-going girls meaning if you use them now as a girl you might end up regretting later in life when the right time comes and you fail to conceive, so it’s not good. —*Nuru, female IDI participant

Challenges such as the lack of female health professionals and difficulty in discussing sexual well-being with health care providers described by one female participant in the quote below also highlighted the need for adolescent-friendly sexual and reproductive health services.

*Where you find your private parts being itchy, you see we were told that if you go the doctor he/she will ask you whether you and your partner used protection and maybe you’ve never even had sex so you will shy away from disclosing it to the doctor about your private parts. (…)around here you find most of these doctors are male and they are not …(…) My opinion is we get to know its name [of the medication] so that when you’re going to buy you know what you are looking for, you have an idea of what to ask for also we should be directed on how to use it, so that when we go a chemist you just buy and go use it. And also prevention measures.* —Nuru, female IDI participant

While some boys reported using condoms and talking more about family planning with their intimate partners following the training, most participants did not discuss safer sex but rather emphasized an aspiration for abstinence to delay or keep off sex completely.

*I have found myself in situations, for instance a girl comes and whines for me and while we dance we get feelings for each other but I tell her that I am not ready to have sex because I know it comes with its consequences like the girl might get pregnant. What would I have done? Again there is HIV/AIDS. Just because of such consequences I choose to back off.* —Martin, male IDI participant

Secondly, boys suggested the need to provide additional support for young men to quit drugs, stating, for example,

*… it is supposed to be like that if you have a problem you can find means to call and tell them am addicted to these what should I do and you help.* —Brian, Male IDI participant

They further highlighted the need for community support systems to sustain the changes that young men make over time and to also teach boys about their rights (as boys may, themselves, be victims rather than perpetrators of sexual violence).

Boys highlighted the need for community support systems to sustain the changes that they make over time and to also teach boys about their rights.

*One thing I would like you to see done … and this thing is always in my mind. Now if, you know most guys who are addicted to drugs are boys who are idle, they are idle, so you find that they start hanging out with people who use drugs. But if this person was busy all the time, it would be nice. So I would love for things to do with sports, try and raise it so that our school can get it (…) you know, those guys, you will find one saying, you know tomorrow I have a game at a certain place, tomorrow I have to be in school by 6. . . . so that person will sleep early and he will not do that. Because he will pause to go and exercise a bit and come back. When he returns, it is late. He takes a shower. Now, you see, there is no way that he will interact with drugs. For him to interact with them, he has to really go out of his way. So things to do with sports, I’d love that to be okay. —*Mathew, male FGD participant

*What I would love to see added is the beacon board. That beacon board means … it means, we as young people … now like the way girls are talked to like girls … this beacon board means that we as young boys are treated as young boys, we are taught that yes, as a young boy, these are your rights. —*Reuben, male FGD participant

Third, in terms of delivery techniques, both boys and girls addressed the need for (additional) refresher classes beyond the current 2 (at 6 months and 1 year) stating that they forgot content and skills over time. They also highlighted the importance of confidentiality, with a few girls requesting that boys should not be allowed in their training sessions (there is one mixed-gender session), and some boys wanting private consultations with the facilitators to get personal advice and support.

*Now that you have come again, don’t get tired of coming back to our school, because, yes you taught us but you know. . . . No one knows it all. . . . you have to keep refreshing us …, So keep coming.* —Lucas, male FGD participant

Finally, participants suggested the expansion of the target population beyond school-going adolescents in urban slums, to reach those out-of-school, including in rural areas. Girls specifically suggested that the training should also target girls who have dropped out of school.

*You forgot to include those girls who have dropped out and also the mothers. (…) you can also talk to them (…) You just look for a meeting point eehhh!* —Sandra, female FGD participant

Additionally, boys noted the importance of involving parents and community elders who can provide more support to young people and parents whom they described to be the “source” of some of the challenges that they face because of stressors that emanate from the home environment or due to parents’ ignorance on how to approach the subject of sexuality with their children:

*… we should also include the elders in the community … so we can know … so they can know our significance and how they can help us, even those of us who are currently in school.—*Charles, male FGD participant

*Ujamaa should …also train our parents because it’s very hard… There are children who live with… single parents, like … like me I live with my mother and brother so it’s hard to find my mother telling me that . . . I stop … abstain from sex, things like that. They should also train parents about. . . . if your child reaches adolescence, you should teach them how these things and these things, how things are …* —Mike, male FGD participant

## DISCUSSION

This qualitative study sought to understand former participants’ experiences of a school-based sexual violence prevention intervention in Nairobi slums that combines ESD training for girls with positive masculinity and bystander training for boys. To our knowledge, this study is the first qualitative exploration of the potential change processes underlying the intervention and of the challenges that adolescents face when they confront norms of violence and gender. Thus, this study is a critical extension of existing impact evaluations.^16^^,^^21^^,^^22^^,^^25^^,^^26^^,^^32^

Consistent with the theory of change, our findings show that even a relatively short, focused intervention can, according to former participants, boost their ability to recognize and resist violence and harmful gender norms and enhance their self-confidence and agency to promote safer and healthier behaviors. Girls who had participated in the intervention reported learning tools to negotiate (potentially) threatening situations, mirroring the experiences of girls and young women participating in ESD trainings in Western countries.^17^^,^^18^^,^^33^ A qualitative evaluation with school-aged girls in New Zealand highlighted how an ESD curriculum enhanced girls’ assertiveness and verbal as well as physical skills to resist sexual assault.^18^ Based on the experiences shared by girls who participated in the current intervention, the results from our study thus indicate that ESD approaches can also benefit school-aged girls in low-income, high-risk settings such as Nairobi slum areas.

Even a relatively short, focused intervention can boost participants’ ability to recognize and resist violence and harmful gender norms, while enhancing their self-confidence and agency.

Our findings add to the growing body of evidence challenging the notion that ESD promotes female victim blaming.^17^^,^^18^^,^^23^^,^^34^^,^^35^ Female participants described feeling *more* empowered as a result of learning about their rights and using verbal and physical self-defense skills, even in cases when doing so was challenging. In a 2018 review, Hollander^17^ stated that such skills remain essential given that “no perpetrator-focused violence prevention strategy has proven effective and where … no strategy could ever provide perfect prevention.” While the responsibility for sexual violence prevention falls on the perpetrator, our results confirm that empowering girls’ agency to refuse unwelcome advances and tackle gender norms that perpetuate a sexual double standard, are necessary (though not sufficient) to disrupt the power of perpetrators.^17^^,^^23^^,^^34^

**Figure:**
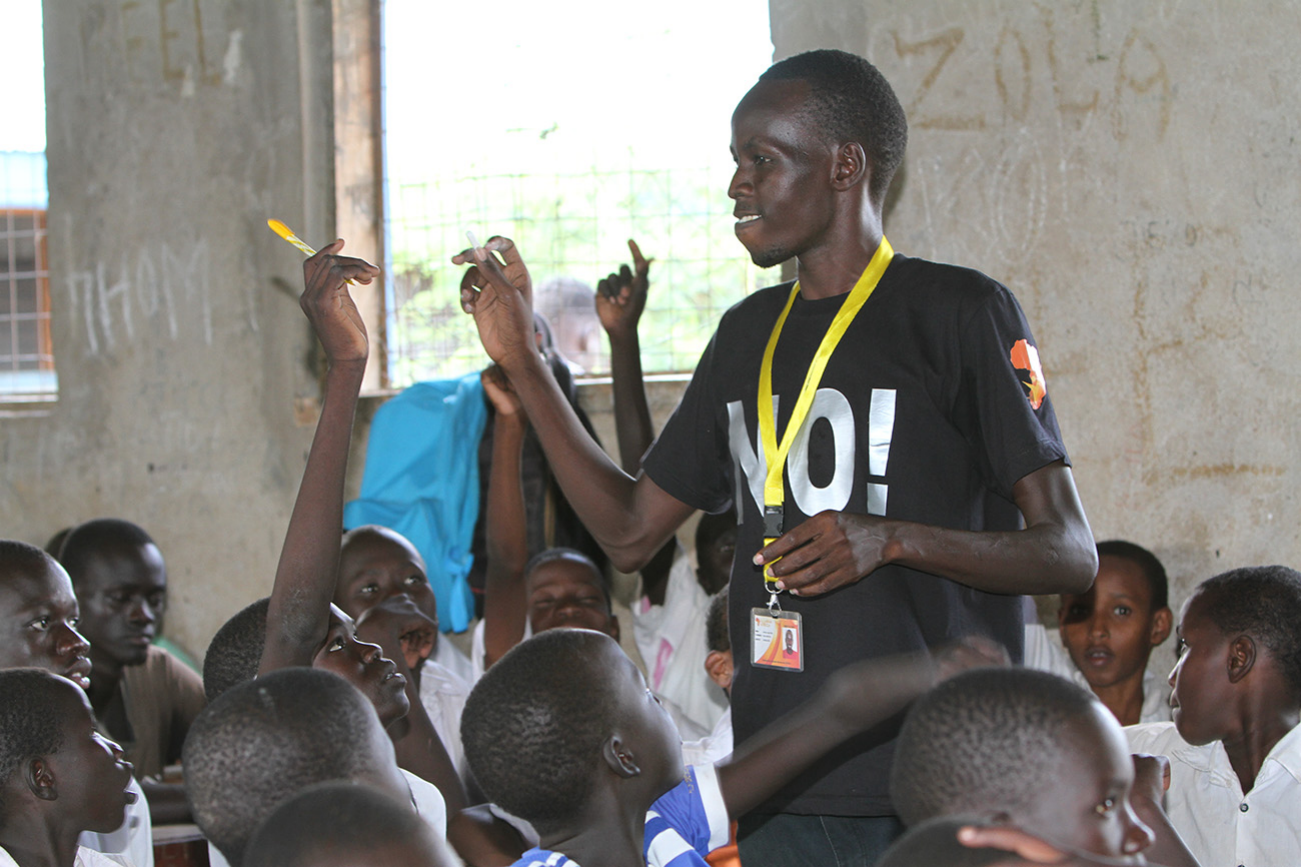
An Ujamaa instructor leading a question and answer session in one of the schools. © 2018 Peter Omondi/Ujamaa Africa

Working with boys and young men is a core, relatively unique component of the intervention. Consistent with evidence from impact evaluations,^25^^,^^26^ male participants described rejecting harmful gender stereotypes and expressed lower tolerance for violence, including reducing risky behaviors that often underlie men’s perpetration of GBV, such as substance abuse and delinquency.^36^^–^^38^ This may be an important contribution of this intervention, given the evidence that many men who perpetrate GBV do so for the first time in their teenage years.^37^ In addition, the challenges that some boys experienced while deciding to leave bad company elucidate the complexities associated with quitting gangs in Nairobi slums,^39^^,^^40^ underscoring the need for post-training support for boys and their communities to ensure that positive life changes are retained.

Our findings also elucidate the complexity of changing gender norms,^41^^–^^44^ with boys emphasizing (more) support for women’s rights while maintaining stereotypical notions, such as female chastity. Furthermore, despite a strong and clear message in the curriculum about sexual consent, narratives from some boys and girls indicate that consent communication in intimate relationships is complex and rooted in norms of token resistance, such as the belief that girls and women say “no” to sex in relationships when they mean “yes.”^45^ It may be that deeply entrenched gender norms limit the application of curriculum lessons on explicit consent communication to platonic and unwanted relations while affecting consent communication in intimate relations appears to be more difficult. This is an important area to consider for future interventions, especially given that boyfriends account for nearly half of the sexual violence perpetrators against young people aged 10–14 in several of Nairobi’s informal settlements.^29^ Indeed, research from high-income countries recommends that interventions to improve sexual consent communication among young men should focus more on sexually assertive communication (saying “yes” rather than “no”).^46^ In addition, one effective ESD program for young women in higher-income settings featured a 3-hour session on relationships and sexuality.^20^

Study findings also elucidate the complexity of changing gender norms and underscore that consent communication in intimate relationships is complex and potentially influenced by gender norms.

As observed in other successful school-based GBV prevention initiatives, the importance of skilled male and female facilitators who use local slang, know their environment, and serve as role models stood out as a key component.^18^^,^^27^ The rigorous selection process and training of local facilitators, much longer than that of other interventions,^27^ and the careful and continuous training process appear to be critical to building trust among and between facilitators and participants.

Finally, we identified several areas for improvement to optimize intervention design and implementation. These include needs to (1) expand the content on sexual and reproductive health and rights (especially regarding debunking myths on contraceptives) and build the knowledge and capacity of facilitators to understanding and deliver comprehensive sexuality education to participants; (2) address boys’ own experiences of sexual violence and other forms of GBV, a reality for many young men and a risk factor for future perpetration^15^^,^^38^; and (3) ensure long-term support after training. In particular, the findings highlight the need to target adolescents’ broader social environments, given that GBV is a complex phenomenon that is shaped by multilevel forces.^14^ The present intervention rests on the individual level, and while it appears to help build participants’ “power with” (e.g., via positive adult relationships), more conscious efforts are needed to directly target communities (parents, teachers, and out-of-school youth) and to enhance and emphasize positive behaviors such as sports as alternatives to risky practices. Many of the underlying risk factors for GBV, including child abuse, witnessing violence in the home, and the socialization of inequitable gender norms, have their roots in adolescents’ homes and communities.^15^^,^^38^ Although building individual skills and challenging gender norms is an integral strategy to end GBV,^15^^,^^24^^,^^37^ global evidence indicates the need to integrate this method into multicomponent approaches to achieve sustainable change.^15^^,^^24^^,^^27^^,^^41^

### Limitations

This qualitative evaluation aimed to explore what participants remembered about the intervention at least 1 year after implementation. While we found that participants did recall the intervention skills and discussions, owing to the retrospective nature of the study, we cannot draw conclusions about the impact of the intervention or treat participants’ experiences as reflective of all girls and boys exposed to the program. It is also possible that the use of interviewers from the same organization that implemented the program led to socially desirable answers; however, it was crucial that the interviewers knew the intervention as well as local communities and slang to gain participants’ trust. We addressed this potential social desirability bias via careful training of the interviewers to solicit both potentially positive and negative experiences, emphasizing the need to learn about challenges encountered by participants. The credibility of our findings is strengthened by the use of a theoretical framework, double-coding of transcripts, and the verification of emerging themes and interpretations with the interviewers.

Despite its limitations, the present study adds novel insights into potential mechanisms of change underlying the effects of interventions aimed at preventing sexual violence in low-income, high-risk communities.

## CONCLUSION

This study fills an important gap in the literature by highlighting participants’ experiences with an empowerment-based, behavioral intervention aimed at preventing sexual violence against adolescents in slums, and by contributing to the call for increased evidence on the intervention^27^ beyond previous impact evaluations.^16^^,^^21^^,^^22^^,^^25^^,^^26^^,^^32^ Our findings suggest several potential pathways through which this relatively short-term intervention can prevent sexual violence by teaching girls skills to recognize and resist harmful situations, and by working with boys to promote positive, nonviolent masculinities and choices. Our analysis also points to several areas of improvement, including the need to incorporate multilevel support structures to target the root causes of sexual violence and other forms of GBV and enable long-term change and sustainable positive behaviors beyond individual skills, which are not sufficient in challenging high-risk environments. Longitudinal mixed-methods studies are needed to unpack how these processes play out over time. In the meantime, our study illustrates how an intervention aimed at both girls and boys early in the life-course can help address the prevailing high rates of sexual violence in Kenya and other low-income settings, an impact made ever more important by how GBV has increased as an indirect result of the COVID-19 pandemic.^47^

## Notes

**Peer Reviewed**

**First published online:** August 27, 2021.
